# An Engineered Probiotic Produces a Type III Interferon IFNL1 and Reduces Inflammations in *in vitro* Inflammatory Bowel Disease Models

**DOI:** 10.1021/acsbiomaterials.2c00202

**Published:** 2022-11-18

**Authors:** Koon Jiew Chua, Hua Ling, In Young Hwang, Hui Ling Lee, John C. March, Yung Seng Lee, Matthew Wook Chang

**Affiliations:** † NUS Synthetic Biology for Clinical and Technological Innovation (SynCTI), National University of Singapore, 117456, Singapore; ‡ Synthetic Biology Translational Research Programme, Yong Loo Lin School of Medicine, National University of Singapore, 117456, Singapore; § Department of Biochemistry, Yong Loo Lin School of Medicine, National University of Singapore,117596, Singapore; ∥ Wilmar-NUS Corporate Laboratory, National University of Singapore, 117599, Singapore; ⊥ Department of Biological and Environmental Engineering, Cornell University, Ithaca, New York 14853, United States; # Department of Paediatrics, Yong Loo Lin School of Medicine, National University of Singapore, 119228, Singapore

**Keywords:** probiotics, *E. coli* Nissle, interferon, anti-inflammation, inflammatory bowel diseases

## Abstract

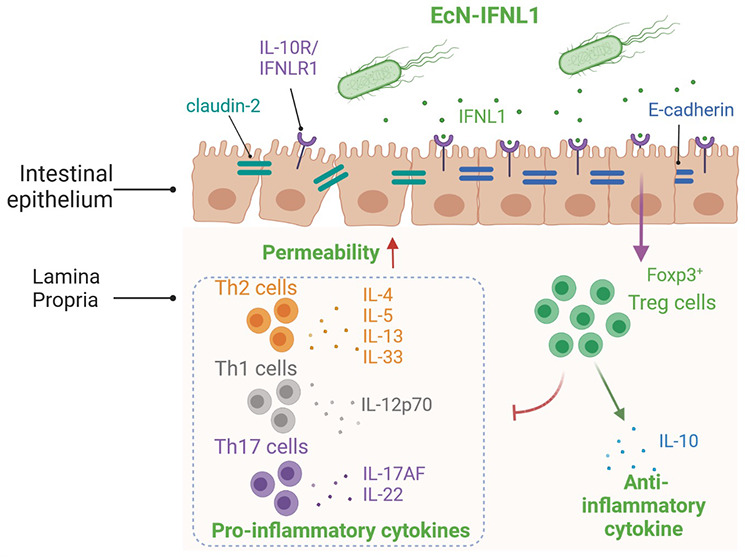

The etiology of inflammatory bowel diseases (IBDs) frequently results in the uncontrolled inflammation of intestinal epithelial linings and the local environment. Here, we hypothesized that interferon-driven immunomodulation could promote anti-inflammatory effects. To test this hypothesis, we engineered probiotic *Escherichia coli* Nissle 1917 (EcN) to produce and secrete a type III interferon, interferon lambda 1 (IFNL1), in response to nitric oxide (NO), a well-known colorectal inflammation marker. We then validated the anti-inflammatory effects of the engineered EcN strains in two *in vitro* models: a Caco-2/Jurkat T cell coculture model and a scaffold-based 3D coculture IBD model that comprises intestinal epithelial cells, myofibroblasts, and T cells. The IFNL1-expressing EcN strains upregulated *Foxp3* expression in T cells and thereafter reduced the production of pro-inflammatory cytokines such as IL-13 and -33, significantly ameliorating inflammation. The engineered strains also rescued the integrity of the inflamed epithelial cell monolayer, protecting epithelial barrier integrity even under inflammation. In the 3D coculture model, IFNL1-expressing EcN treatment enhanced the population of regulatory T cells and increased anti-inflammatory cytokine IL-10. Taken together, our study showed the anti-inflammatory effects of IFNL1-expressing probiotics in two *in vitro* IBD models, demonstrating their potential as live biotherapeutics for IBD immunotherapy.

## Introduction

Inflammatory bowel diseases (IBDs) are a collective term for various pathological subtypes of intestinal epithelium inflammation. Any part of the small and large intestines can be inflamed, and inflamed tissue can develop into intestinal cancer if left untreated.^1^ Healthy intestines house trillions of commensal microbes that aid in digestion and influence the development and function of the host’s mucosal immune system. The intestinal epithelium functions as a physical and biochemical barrier between the host and these microbes. Epithelium inflammation can disrupt the host–microbe barrier, causing intestinal barrier dysfunction when the tight junctions of epithelial cells are disrupted. The disrupted tight junction allows uncontrolled paracellular transport of sodium, potassium, and fluid, contributing to diarrhea and increasing the risk of infection to the host along with microbial translocation into the bloodstream.^2^ Ulcerative colitis (UC) and Crohn’s disease (CD), the most common IBDs, are typically attributed to the imbalance of T helper 2 (Th2)-associated cytokines in UC and Th1-associated cytokines in CD.^3^

Currently available treatments for IBDs are multifaceted and aim to control the disease by regulating the patient’s immune system.^4^ The most common immunotherapy in IBDs involves designing antibodies to target pro-inflammatory cytokines implicated during IBD development, such as TNFα and IL12/23.^5^ While these antibody treatments can remit IBD initially, long-term usage of such therapies may increase the risk of opportunistic infections.^6^ Accordingly, the supplementation of specific anti-inflammatory cytokine IL-10 is an alternative means to reverse the inflammatory environment. However, its clinical trials in CD patients have so far been disappointing.^7,8^ Thus, we hypothesized that, instead of targeting a specific cytokine or pathway, the inflammatory IBD environment can be more effectively reversed by modulating the expression of Th1- and Th2-associated cytokines.

Type III interferons (IFN-III), including interferon lambda 1 (IFNL1 or IL-29), have protein sequences and structures highly similar to IL-10.^9^ IFN-III are associated with immunomodulatory effects in various diseases, including autoimmune diseases, viral infections, and cancer.^10^ For instance, IFN-III administration is reported to reduce the expression of Th2-associated cytokines such as IL-13 in mouse models of autoimmune diseases. The reduced expression of IFNL1 and IFNL2 was observed in asthma^11^ and autoimmune arthritis,^12^ respectively. IFN-III signals through a heterodimeric receptor complex consisting of its own unique IFNL1 receptor 1 (IFNLR1) and a shared IL-10 receptor subunit 2 (IL10R2).^13^*IFNLR1* displays restricted cellular expression and is found mostly on epithelial cells with the highest expression found in the intestinal epithelial cells.^14^ The downstream signaling pathways activated by IFN-III are similar to but nonredundant to that of type I IFN (IFN-I). Due to the restricted expression of *IFNLR1*, it is expected to exhibit fewer side effects when used as a target of therapeutics, compared to IFN-I, whose receptors are ubiquitously expressed.^15^ Furthermore, previous studies that used IFN-I as therapeutics to treat IBD have demonstrated disappointing effects in both animal and clinical trials, mostly due to unwanted reactions.^16^

Aside from being a member of the IL-10 superfamily, IFNL1’s ability to signal via various pathways involving IL-10 and type I IFNs, makes it a promising candidate to be investigated for IBD therapy.^17,18^ Various studies have demonstrated an increase of anti-inflammatory cytokines including IL-10 in the blood serum of IBD patients.^19−21^ However, several immunotherapies developed to target a specific pro-inflammatory cytokine or pathway have been unsuccessful to date.^22^ Notably, IFN-III can accelerate intestinal mucosal healing and epithelial regeneration^23,24^ and exert protective effects on the intestinal epithelial barrier by defending against opportunistic pathogens.^25^ Given the versatility of IFN-III’s immunomodulation, live microbes could be engineered and used to deliver IFN-III-mediated therapeutic effects against various diseases including IBD.^26,27^ In addition, drug delivery by live microbes carries unique advantages such as stability and specificity.

In this study, a probiotic strain *Escherichia coli* Nissle 1917 (EcN) was engineered to promote anti-inflammatory effects via IFNL1. EcN, a robust Gram-negative probiotic commonly used for treating gut disorders and diseases such as UC,^28^ is an attractive drug delivery system. Briefly, we engineered probiotic EcN to produce and secrete IFNL1 under the control of a nitric oxide (NO)-inducible promoter in the plasmid (EcN-IFNL1) and chromosome (EcN-gIFNL1). NO is a biomarker for intestinal inflammation,^29^ and an NO-controlled delivery of therapeutics agent for CD was previously explored.^30^ Next, we validated the anti-inflammatory effects of the engineered EcN strain in two *in vitro* IBD models.^31^ EcN-gIFNL1 suppressed the production of various pro-inflammatory cytokines, enhanced anti-inflammatory cytokine production, and promoted the population of regulatory T cells. Moreover, EcN-gIFNL1 rescued the integrity of the inflamed epithelial cell monolayer. Our study therefore demonstrates the potential for developing live biotherapeutics for IBD immunotherapy.

## Materials and Methods

### Plasmids, DNA Constructs, and Oligonucleotides

All plasmids were constructed according to standard restriction cloning procedures. Codon-optimized human *IFNL1* (*IFNL1*) was synthesized by IDT (Singapore). *YebF* was PCR amplified from the TOP10 genome, while promoter pNorV was PCR amplified from the EcN genome, using Kapa HiFi polymerase (Kapa Biosystems). The promoters pNorV, RBS j23100, *YebF*, and *IFNL1* were cloned into the pUC18 vector, resulting in recombinant plasmids including pUC18-pNorV-YebF. Similarly, pUC18-pNorV-YebF-GFP was also constructed. A gene cassette comprising the FRT-flanked kanamycin resistance gene (*kanR*), pNorV-YebF-IFNL1, and lacI/lacZ homologous regions was amplified using Q5 polymerase (New England Biolabs) and cloned into pUC18, resulting in gene cassettes for genomic integration.

### Integration of IFNL1 Expression Cassette into EcN Genome

The pNorV-YebF-IFNL1 cassette was knocked into the lacI/lacZ domain in the EcN genome using phage λ Red recombinase as described by Datsenko and Wanner.^32^ Briefly, an approximately 3kb fragment consisting of the FRT-flanked *kanR*, pNorV-YebF-IFNL1, and lacI/lacZ homologous regions was amplified and transformed into EcN expressing λ Red recombinase.^33^ Transformants were then grown on LB agar supplemented with IPTG (40 μg/mL), X-Gal (2 μg/mL), and kanamycin (50 μg/mL) at 37 °C. White colonies were selected, and the knock-in sequence was verified by colony PCR. Positive colonies carrying pNorV-YebF-IFNL1 were made electro-competent and transformed with pCP20, an ampicillin resistance vector that carries a temperature-sensitive replicon and allows for the thermal induction of FLP synthesis. Positive transformants were then selected on LB agar with added 100 μg/mL ampicillin at 30 °C and purified nonselectively at 43 °C before being tested for the complete loss of antibiotic resistance. The obtained colonies were confirmed by PCR using genome DNA as a template. The confirmed strain was named EcN-gIFNL1. A control strain chromosomally integrated with pNorV-YebF was constructed using the same method.

### Cell Lines and Culture Conditions

Human colon epithelial cells (Caco-2, ATCC HTB-37) were maintained in Dulbecco’s modified Eagle medium (DMEM, Gibco, ThermoFisher) supplemented with 15% fetal bovine serum (FBS, Biowest, France) and 1% penicillin/streptomycin (Sigma-Aldrich). T cells Jurkat (ATCC TIB-152) were maintained in RPMI 1640 medium (Lonza) supplemented with 10% FBS and 1% penicillin/streptomycin. All cells were incubated at 37 °C in a humidified 5% CO_2_-containing atmosphere.

For the FITC-dextran permeability assays and Western blot analysis, Caco-2 cells were seeded at a density of 7.5 × 10^4^ cells per well in 24-well transwells (Millipore) for 5 d, after which they were subjected to polarization. Polarization was achieved by culturing the cells in FBS-free DMEM on the apical compartment, while FBS-supplemented DMEM was provided on the basal compartment, for at least 14 d. The medium was changed to both apical and basal sides every 2 d.

To induce inflammation, a cocktail of pro-inflammatory cytokines (interleukin 1-beta (rhIL-1β, 25 ng/mL), tumor necrosis factor alpha (rhTNF-α, 50 ng/mL), interferon gamma (rhIFN-γ, 50 ng/mL), and lipopolysaccharide (LPS, 1 μg/mL)^34^) were supplemented in low glucose (1 mg/mL) DMEM without antibiotics and FBS and added to the apical compartment. In the basal compartment, 2 × 10^5^ cells/mL of Jurkat T were seeded in RPMI as described above. For 24 h, inflammation was carried out, followed by 8 h treatments where 10 mM Mesalazine (Sigma-Aldrich), 100 ng/mL rhIFNL1, and 10^7^ cells/mL EcN-WT, EcN-YebF, EcN-IFNL1, EcN-gYebF, or EcN-gIFNL1 were applied to the apical compartments. Caco-2 cells, Jurkat T cells, and culture supernatants were collected and analyzed. Recombinant human IFNL1 (rhIFNL1), IL-1β, TNF-α, and IFN-γ were purchased from R&D Systems. The LPS was purchased from Sigma-Aldrich.

### Primary Cells and Culture Conditions

Primary intestinal epithelial cells (InEpC) and myofibroblasts (InMyoFib) (Lonza) were maintained in Smooth Muscle Growth Basal Medium (SmBM) supplemented with insulin, hFGF-B, hEGF, 5% FBS, and 0.1% gentamycin. A 3D scaffold structure of InMyoFibs and InEpCs was created according to the manufacturer’s protocol. InMyoFibs (10^5^ cells/cm^2^) from the third passage (P3) were seeded onto the basal side of each transwell 1 d before InEpCs (Lonza) seeding. InEpCs were freshly thawed and cultured at a density of 10^5^ cells/cm^2^ on the apical compartment precoated with 30 μg/mL rat tail type I collagen (Sigma-Aldrich). InEpC, InMyoFib, SmBM, and the medium supplements were purchased from Lonza. All cells were incubated at 33 °C in a humidified 5% CO_2_ atmosphere.

CD4^+^ T cells were isolated from human peripheral blood mononuclear cells (PBMCs, ATCC PCS-800-011) using an EasySep Human CD4^+^ T Cell Isolation Kit according to the manufacturer’s protocol. Isolated CD4^+^ T cells were maintained in ImmunoCult XF medium supplemented with ImmunoCult Human CD3/CD28/CD2 T Cell Activator and 600 IU/mL hIL-2 (R&D Biosystems) and incubated at 37 °C in a humidified 5% CO_2_ atmosphere until the cell count reached approximately 10^7^ cells/mL. The cells were then cryopreserved in CryoStor CS10 preservation medium until further use. Immunocult XF medium, T cell activator, and CryoStor CS10 were purchased from StemCell Technologies.

Four hours before the onset of inflammation, the medium was changed to fresh SmBM medium in the apical compartment, while 2 × 10^5^ cells/mL of the isolated CD4^+^ T cells supplemented with hIL-2 were added to the basal compartment. Inflammation was induced for 36 h as described above. Treatments with 10^7^ cells/mL EcN-gYebF or EcN-gIFNL1 in the apical compartments were carried out for 10 h before the cells and supernatant in both compartments were collected for further analysis. A diagram showing the coculture setup is shown in Figure S1A. All cell culture media used in coculture studies were free of antibiotics.

### RNA Extraction and Real-Time PCR

Total RNA was extracted from treated Caco-2 cells, Jurkat T cells, and IECs using TRIzol (Invitrogen), and 1 μg of total RNA was reverse transcribed using qScript cDNA Supermix (Quantabio, USA). Real-time PCR was carried out using the Luna Universal qPCR Master Mix (New England Biolabs) and performed on the CFX Connect Real-time PCR Detection System (Biorad). All procedures were performed according to the manufacturers’ protocols. The primers used are iNOS_F: ACCTCCAGTCCAGTGACACA; iNOS_R: AATCCCTTTGGCCTTATGGT; Foxp3_F: AACAGCACATTCCCAGAGTTCCT; Foxp3_R: CATTGAGTGTCCGCTGCTTCT; GAPDH_F: GCTCTCTGCTCCTCCTGTTC; GAPDH_R: AAATGAGCCCCAGCCTTCTC.

### Protein Extraction and Western Blot

Caco-2 cells were carefully removed from the membranes with 100 μL of protein extraction buffer (0.1% Triton-X) by repeatedly pipetting up and down. The cell suspension was then centrifuged at 10,000 rpm, 4 °C for 10 min, and the lysate was transferred to a clean new tube. The protein concentration in each sample lysate was measured using a NanoDrop UV–vis spectrophotometer at 280 nm. The same amount of each sample was removed, mixed with 6× loading buffer, and denatured at 100 °C for 5 min before applying to a gradient (10–15%) SDS-PAGE gel. Proteins were then transferred to a 0.45 μm pore size nitrocellulose membrane (GE Healthcare) before blotting for different tight junction proteins. The SignalFire ECL reagent (Cell Signaling Technology) was used to detect the protein bands, and the bands were visualized with the Amersham Imager (GE Life Sciences). Anti-E-cadherin, anticlaudin-2, antitricellulin, and antibeta-actin (Cell Signaling Technology) antibodies were used to detect the respective proteins. All antibodies were purchased from Life Technologies unless otherwise stated.

### Cryopreservation, Immunofluorescence, and Confocal Microscopy

The membrane from each transwell was removed using a scalpel and briefly washed with sterile 1× PBS. The membranes were first treated with 4% paraformaldehyde (PFA) for 10 min at 4 °C and washed thrice with 1× PBS before treatment with 30% sucrose for 15 min. The membrane was then incubated overnight at 4 °C in a 30% sucrose solution. The membranes were then removed and incubated in 30% sucrose:OCT (1:1) for at least an hour at room temperature and finally incubated in 100% OCT for 15 min. Each membrane was then embedded in a vertical position in 100% OCT on a cold metal plate on dry ice. Embedded membranes were stored at −80 °C until sectioning. Six μm sections were made from each membrane using the Cryostar NX50 cryostat (ThermoFisher). The sections were placed on poly-l-lysine coated glass slides and fixed with 4% PFA for 5 min before being washed with HBSS thrice (Figure S1B). Labeling with AlexaFluor 644 wheatgerm agglutinin (WGA, 5 μg/mL, ThermoFisher) was carried out for 10 min at room temperature. The slides were washed thrice with HBSS, followed by blocking with 0.1% BSA for 30 min at room temperature. After this, the slides were coincubated with anti-E-cadherin and anticlaudin-2 antibodies overnight at 4 °C. Samples were then incubated for 1 h at room temperature with the FITC-labeled secondary antibodies (Cell Signaling Technology) and then stained with the nuclear stain 4′,6-diamidino-2-phenylindole (DAPI, NucBlue Fixed Cell ReadyProbes Reagent, LifeTech) before a clean coverslip was mounted over each sample. All incubation steps were carried out on a rotator. Samples were gently washed with 0.1% TBST. Prolong Gold antifade mountant (LifeTech) was used to preserve the fluorescence in each sample. Confocal microscopy was carried out using an Olympus FV3000 confocal microscope. The relative fluorescence intensity of claudin-2 (green) or E-cadherin (green) in the region of interest (ROI) was obtained by quantifying the corresponding signal and normalizing it against the WGA (magenta) signal using ImageJ software ver. 1.53.

### FITC-Dextran Permeability Assay

Supernatants from both the apical and basal compartments were removed, and 1 μg/mL of 10 kDa FITC-dextran (FD10) was diluted in fresh cell culture medium and applied to the apical compartment at the end of each indicated treatment. Sterile 1× PBS was supplemented to the basal compartment, and the culture was incubated at 37 °C in a humidified 5% CO_2_ atmosphere for 1 h before 100 μL of the supernatant was removed from both the apical and basal compartment. Fluorescence intensities were determined at an excitation wavelength of 485 nm and an emission wavelength of 525 nm using a Synergy H1 microplate reader (BioTek).

### Flow Cytometry

Cells in the basal compartments in all coculture setups were stained with surface markers FITC-labeled anti-CD4 (Cell Signaling Technology), APC-labeled anti-CD25 (Cell Signaling Technology), PE-Cy7-labeled anti-CD127 (eBioScience), and Treg-specific transcription factor PE-labeled anti-FoxP3 (eBioscience) antibodies for identification of Treg cells. The eBioscience Foxp3/Transcription Factor Staining Buffer Set was purchased from ThermoFisher. Single cell suspensions were resuspended at 1 × 10^6^ cells/mL and were initially stained with the surface markers for at least 30 min in 4 °C. Cells were then fixed and permeabilized. Intracellular staining of Foxp3 was then carried out according to the manufacturer’s protocol. Flow cytometry was performed with BD LSR Fortessa, and the results were analyzed using the Flowjo software.

### ELISA and Multiplex Assay

ELISA was carried out for the detection of secreted IFNL1 by the engineered cells. Anti-IFNL1 capture antibodies (4 μg/mL) (ThermoFisher) were coated on 96-well MaxiSorp plates (ThermoFisher) overnight at 4 °C. All wells were then blocked with 0.1% BSA for 1 h at room temperature. Samples or IFNL1 standards prepared using rhIFNL1 (R&D Systems) were loaded in triplicates and incubated at 4 °C for overnight. Anti-IFNL1 detection antibodies (2 μg/mL) (R&D Systems) and subsequently secondary antimouse antibodies (Cell Signaling Technology) were added to each well and incubated at room temperature for 1 h in a stepwise manner. All antibodies were diluted in 0.1% BSA blocking buffer. Wells were washed with 1× PBS three times and blotted by patting the plate against a paper towel between every step. TMB substrate (Pierce) was added to each well and allowed to sit in the dark for 10 min, and the reaction was stopped with sulfuric acid. Quantitative readings of the absorbance at 450 nm were carried out using a Synergy H1 microplate reader. Multiplex assays of coculture supernatants were analyzed using customized ProcartaPlex plates (ThermoFisher). All steps were carried out according to the manufacturer’s protocol.

## Results and Discussion

### Production and Secretion of IFNL1 by EcN-IFNL1 in Response to Nitric Oxide

**Figure 1:**
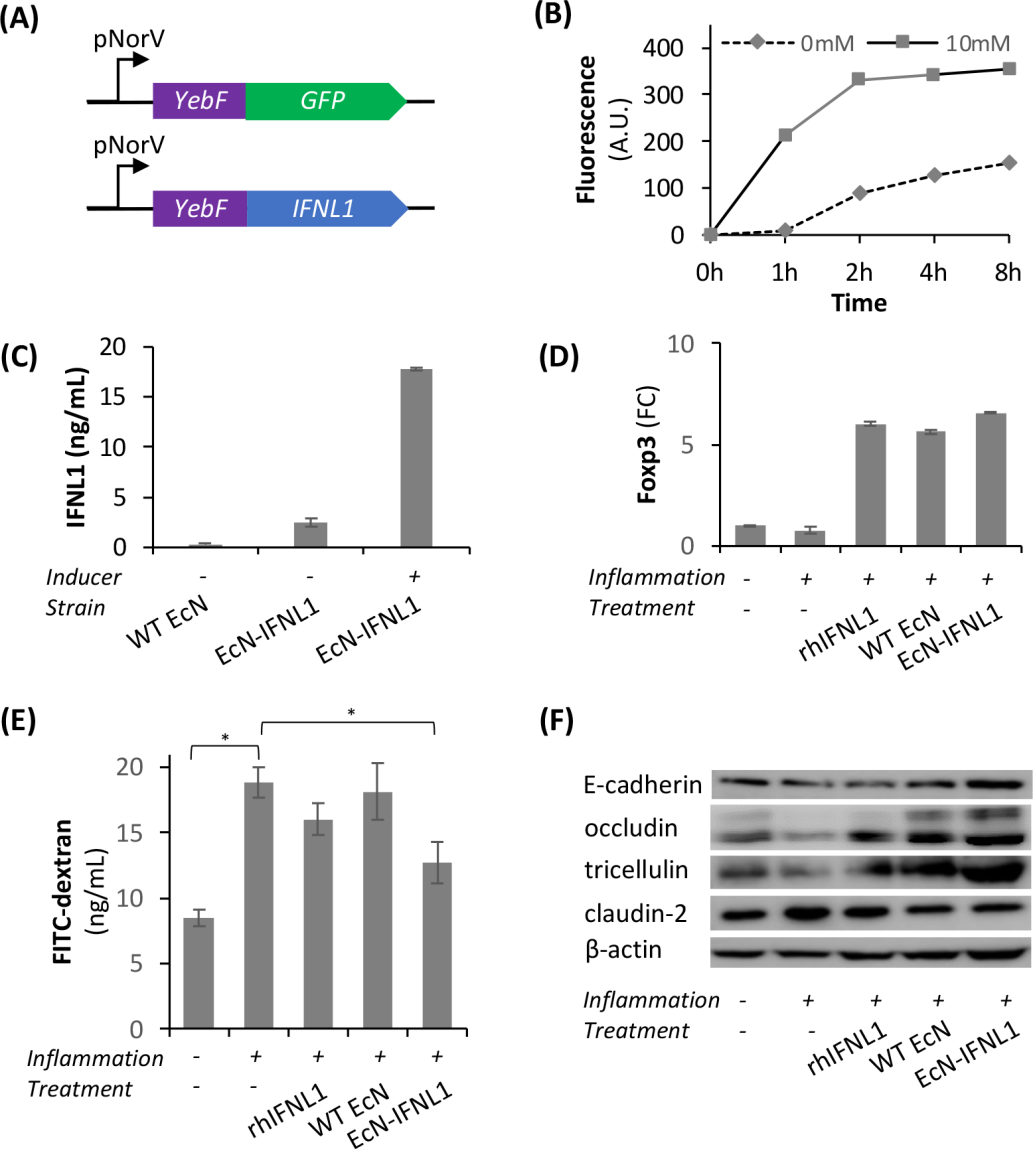
Functional characterization of EcN-IFNL1 expressing and secreting IFNL1. (A) Schematics of pNorV-controlled expression of secretion tag YebF-fused GFP or IFNL1. (B) The fluorescence intensity of GFP secreted by the transformed EcN was determined with (10 mM) and without (0 mM) inducer SNP. (C) The amount of IFNL1 produced and secreted by the engineered EcN was determined by ELISA. Inducers were supplemented as indicated. *n* = 3 biological repeats. (D) Transcription levels of Treg cell transcription factor, Foxp3, in Jurkat T cells were normalized to the transcription levels of housekeeping gene *GAPDH* and determined by real-time PCR. FC, fold change. (E) Permeability of the Caco-2 epithelial layer determined by the FITC-dextran assay. (F) Western blot showing the differential expressions of the various tight junction proteins of Caco-2 cells with the indicated treatments for 8 h. *n* = 6 biological repeats. *, *p* < 0.05 (Student’s *t* test).

In this study, we selected a probiotic bacterial strain EcN and engineered it to produce and secrete IFNL1 in a controllable manner. To this end, we first constructed a genetic circuit to produce and secrete green fluorescent protein (GFP) in the presence of NO, an inflammation marker observed in the gut. Specifically, *GFP* fused with a Gram-negative bacterial secretion tag *YebF* was cloned under the control of an NO-inducible promoter pNorV in pUC18 (Figure 1A).^35^ pNorV activation is regulated by the endogenous regulator, NorR, which in turn reacts with NO and reactive nitrogen intermediates (RNIs).^36^ The resulting plasmid pUC18-pNorV-YebF-GFP was introduced into EcN, resulting in the strain EcN-GFP. To confirm the inducible production and secretion of GFP in EcN-GFP, we inoculated EcN-GFP cells at 10^7^ CFU/mL in phenol red-free DMEM medium supplemented with sodium nitroprusside (SNP), a source of NO, and evaluated cell densities, GFP induction, and secretion. Figure S1A shows that the cell density of EcN-GFP with ≤5 mM SNP was comparable to EcN-GFP without SNP and that the density decreased by 20% (10 mM SNP) and 90% (30 mM SNP). There was relatively low growth inhibition and high nitrite (14 μM) produced at 8 h (Figure S2) at 10 mM SNP. Hence, we chose to use 10 mM SNP to induce the IFNL1 expression for subsequent functional assays. Figure 1B shows that the fluorescence intensity of the supernatant collected from EcN-GFP+10 mM SNP was over 2-fold higher than EcN-GFP without SNP, suggesting the successful induction of GFP production and secretion in the presence of the inducer SNP. These results confirmed the induction of GFP expression by SNP and its secretion mediated by the YebF secretion tag.

Following the confirmation of SNP-inducible GFP production and secretion, we replaced *GFP* with *IFNL1*. To determine the amount of IFNL1 secreted, ELISA was carried out. Briefly, wild-type (WT) EcN or EcN-IFNL1 were inoculated at 10^7^ CFU/mL for 8 h. Approximately 17 ng/mL of IFNL1 was detected in EcN-IFNL1 cultures supplemented with 10 mM SNP, while less than 5 ng/mL of IFNL1 was detected in the EcN-IFNL1 culture in the absence of SNP (Figure 1C). As expected, WT-EcN showed no observable IFNL1 expression. This result suggests the successful production and secretion of IFNL1 as induced by 10 mM SNP.

### Anti-inflammatory Effects of EcN-IFNL1 in Caco-2/Jurkat T Cell Coculture Model

We then proceeded to test the functionality of EcN-IFNL1 using a coculture model consisting of Caco-2 (apical compartment) and Jurkat T cells (basal compartment). An inflammatory cocktail was added to the coculture 24 h prior to EcN-IFNL1 treatment. For a clearer comparison, we included controls with the treatments of either EcN-WT or 10 mM mesalazine, a first line drug for UC. We first sought to confirm NO upregulation in the inflamed coculture model by determining the expression of induced nitric oxide synthase (iNOS), an enzyme which generates NO and is an indicator of inflammation.^37^Figure S3 shows that *iNOS* was upregulated by 200-fold in the inflamed Caco-2 cells cocultured with Jurkat T cells in the basal compartment. This result suggests that our inflammation model produced NO, which served as an inducer for the engineered EcN to express and secrete IFNL1.

Given the role of IFNL in the expansion of CD4^+^CD25^+^Foxp3^+^ regulatory T (Treg) cell population,^11,38^ we were interested in whether *Foxp3* and pro-inflammatory cytokines expressions are changed after treatment with our engineered EcN that expressed IFNL1 (EcN-IFNL1). When inflammation was induced in a coculture model consisting of Caco-2 cells and Jurkat T cells, the transcription level of *Foxp3* in Jurkat T cells moderately decreased. When treated with the rhIFNL1 protein, EcN-WT, or EcN-IFNL1, the transcription level of *Foxp3* increased over 6-fold compared to the untreated group (Figure 1D). Following the confirmation that *Foxp3* transcription was enhanced, we determined the changes in pro-inflammatory cytokines (IL-4, -13, -33, -12). Figure S4A shows that pro-inflammatory cytokines related to both Th1 (IL-12) and Th2 cells (IL-4, -13, -33) increased up to 92-fold when the coculture models were inflamed. EcN-IFNL1 treatment significantly reduced the production of these pro-inflammatory cytokines compared to the controls (no treatment, mesalazine, EcN-WT) (Figure S4B–E). These results confirmed the upregulation of Foxp3 expression in EcN-INFL1 and the downregulation of pro-inflammatory cytokines (IL-12, -4, -13, and -33) with EcN-IFNL1 treatment, suggesting inflammation amelioration.

To confirm the physiological effects on the inflamed epithelial cells with or without EcN-IFNL1 treatment, we carried out an FITC-dextran assay to analyze the permeability of the Caco-2 epithelial layer. There was an increase of FD10 concentration (FC 2.2) in the basal compartment of the inflamed and untreated model compared to the uninflamed control (Figure 1E). No significant reduction in the concentration of FD10 was detected in the basal compartment of cocultures treated with either rhIFNL1 or EcN-WT compared to the uninflamed model. EcN-IFNL1 treatment gave a significant reduction of FD10 in the basal compartment, indicating a tighter epithelial layer or less permeability (Figure 1E). Next, we performed a Western blot to determine whether EcN-IFNL1 treatment influenced tight junction protein expression. Figures 1F and S5 show that inflammation of the cocultures reduced the expression of the tight junction proteins (i.e., E-cadherin, occludin, and tricellulin) while also increasing the expression of the ion channel protein claudin-2. EcN-IFNL1 treatment increased the expression of tight junction proteins and reduced claudin-2 expression (Table S1), which is consistent with the results of FD10 measurements. Therefore, our results suggest EcN-IFNL1 improves tight junction integrity in inflamed Caco-2 cells.

### Anti-inflammatory Effects of EcN-gIFNL1 in Primary Epithelial Cells

**Figure 2:**
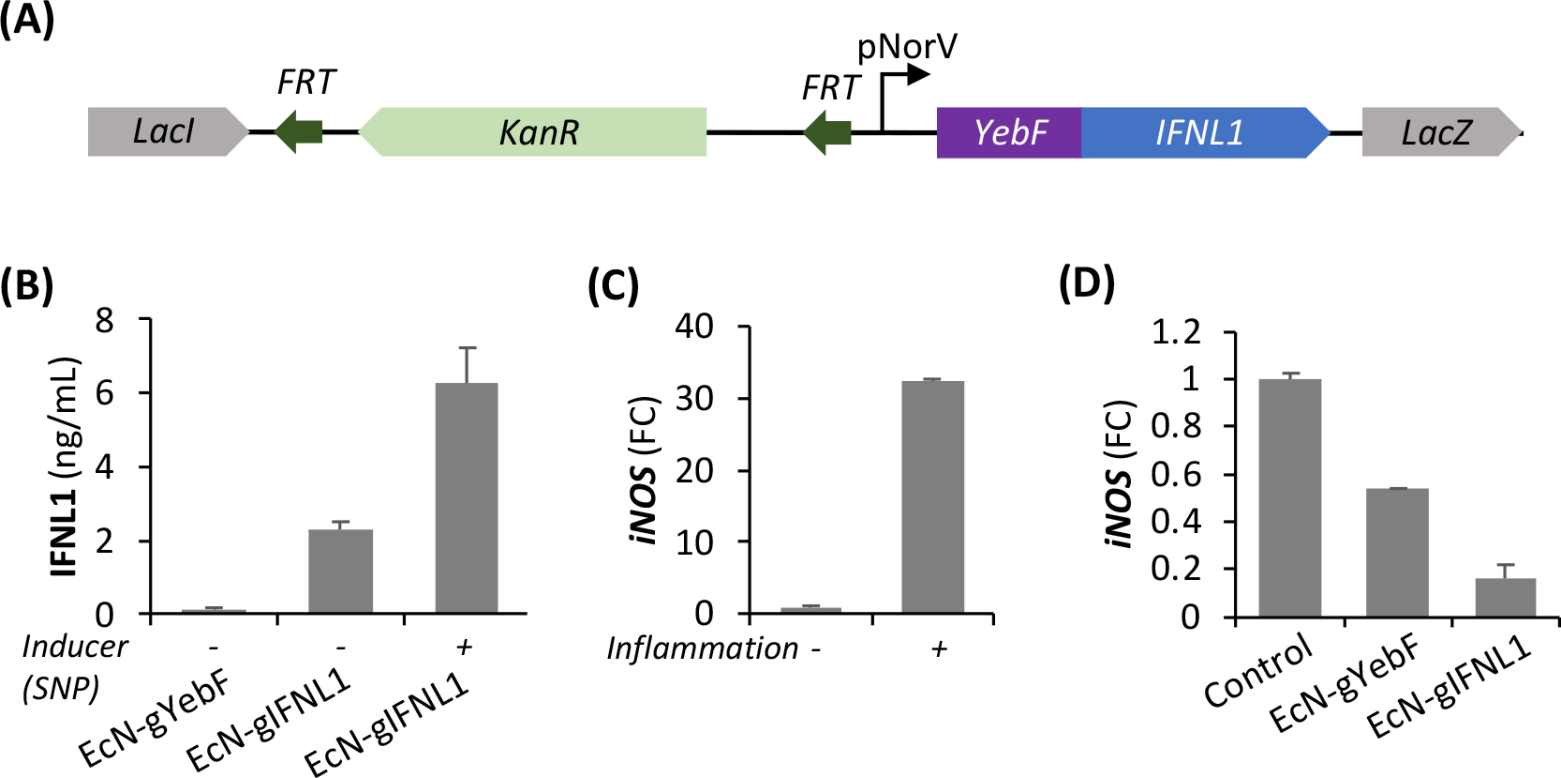
Chromosomal expression and secretion of IFNL1 and amelioration of inflammation by EcN-gIFNL1. (A) Schematics of the IFNL1 production-secretion cassette for genomic integration. *KanR* was used as the selection marker gene for successfully integrated clones. (B) The concentration of secreted IFNL1 by EcN-gIFNL1 as determined by ELISA. (C) Induced NO synthase (iNOS) expression was confirmed in inflamed primary intestinal epithelial cells (IECs). (D) iNOS expression was significantly downregulated by EcN-gIFNL1. FC, fold change.

After demonstrating the anti-inflammatory effects of EcN-IFNL1 with plasmid-based *IFNL1* expression, we sought to develop EcN as a chassis that stably delivers IFNL1. To this end, we integrated the IFNL1 production-secretion cassette into the EcN genome using a lambda red recombinase-based method (Figure 2A).^32^ Particularly, we chose the nonessential lacI-lacZ region as the integration site and used *KanR* as a marker gene for selecting colonies that carried the IFNL1 production-secretion cassette. We confirmed the colonies by genomic PCR and named the obtained strain EcN-gIFNL1 (data not shown). Next, we induced IFNL1 production by adding 10 mM SNP and measured IFNL1 concentration in the supernatant. Figure 2B shows that IFNL1 at 6 ng/mL in the presence of inducer SNP was detected in the supernatant of EcN-IFNL1 at an amount 3-fold higher than that in uninduced EcN-IFNL1. Our results suggest that the engineered EcN carrying a genomic copy of the IFNL1 production-secretion cassette (EcN-gIFNL1) successfully produced IFNL1 upon SNP induction and secreted IFNL1 into the supernatant mediated by the YebF secretion tag.

Given upregulation of *iNOS* expression in inflamed Caco-2 cells cocultured with Jurkat T cells (Figure S3), we were interested in the effect of EcN-gIFNL1 treatment on *iNOS* expression levels in inflamed primary intestinal epithelial cells (IECs). To mimic a colorectal environment, we set up a scaffold-based 3D coculture model, where we seeded IECs on the membranes of transwells (“apical/epithelial”), and cultured myofibroblasts and T cells in the basal compartment (“basal/lamina propria”) followed by RT-PCR analysis of *iNOS* expression. Figure 2C shows that the transcription level of *iNOS* was increased by 32.4-fold in the inflamed IECs over the uninflamed IECs. Figure 2D shows that the *iNOS* transcription level was reduced by 84.1% in the inflamed IECs treated with EcN-gIFNL1, significantly higher than the EcN-gYebF treatment (46.5%). The reduction of the *iNOS* expression level in the inflamed primary IECs with EcN-gIFNL1 treatment suggests that EcN-gIFNL1 mediated anti-inflammatory effects.

### Effects of EcN-gIFNL1 on Induced and Effector Treg Cell Populations

Given the increase of *Foxp3* transcription in the inflamed Jurkat T cells treated with EcN-IFNL1 (Figure 1D), we hypothesized that EcN-gIFNL1 treatment could promote the differentiation of induced Treg (iTreg) cell populations under inflammation. As the name suggests, iTreg cells arise from peripherally circulating naïve CD4^+^ T cells upon certain stimulation, distinguishing them from the naturally occurring Treg cells (nTregs) that develop from progenitor cells in the bone marrow.^39^ We induced inflammation in CD4^+^ T cells isolated from human peripheral blood mononuclear cells (PBMCs) and treated CD4^+^ T cells with rhIFNL1, EcN-gYebF, or EcN-gIFNL1 by applying the bacterial culture on a transwell. Next, we collected and analyzed CD4^+^ T cells with anti-CD4, -CD25, -CD127, and -Foxp3 by flow cytometry. Our results (Figures S6 and 3A) show that, under inflammation, EcN-gIFNL1 treatment resulted in an iTreg cell population of 14.6% of the total lymphocytes, which is 6% higher than rhIFNL1 treatment (8.6%), 2.8% higher than EcN-gYebF treatment (11.8%), and 3.5% higher than the negative control without inflammation. These changes are statistically significant and support our hypothesis of EcN-gIFNL1 driving the increase in iTreg cell population.

**Figure 3:**
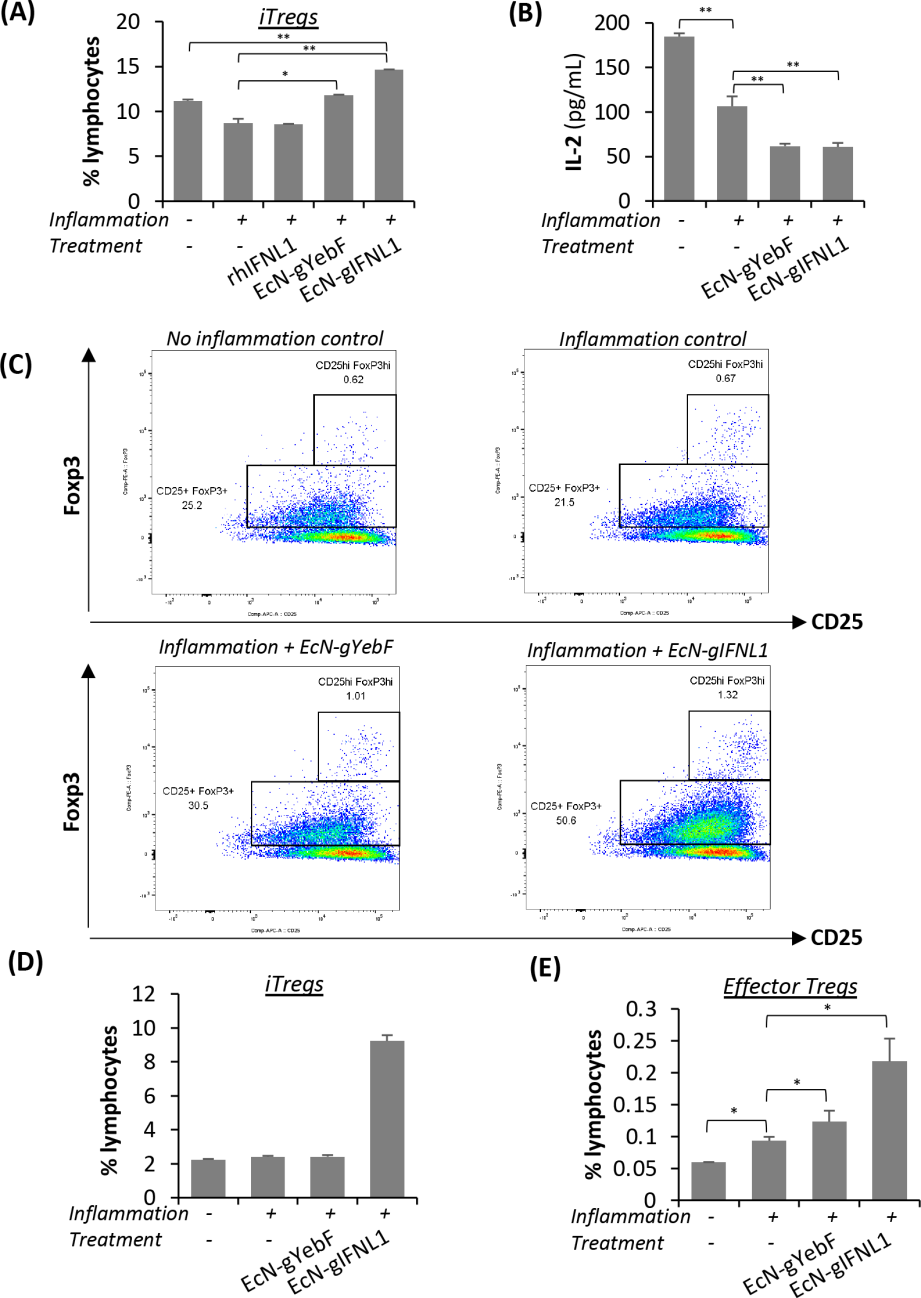
Effect of EcN-gIFNL1 on regulatory T (Treg) cell populations. Flow cytometric analysis showed a synergistic effect of EcN-gIFNL1 on enhancing the population of CD4^+^CD25^+^Foxp3^+^ induced Treg (iTreg) cells. (A) Naïve CD4^+^ T cells were cultured for 24 h under inflammatory conditions and treated for 10 h with the indicated treatments. (B) Naïve CD4^+^ T cells were cocultured with primary IECs under inflammatory conditions and subsequently treated for 10 h with the indicated treatments. The concentration of IL-2 in the cell supernatants was analyzed through a multiplex assay. Similarly, flow cytometric analysis of the T cells in the coculture model revealed a synergistic effect of EcN-gIFNL1 on the enhancement of Treg cells. (C) Plots of iTreg cells (CD25^+^Foxp3^+^) and effector Treg cells (CD25^hi^Foxp3^hi^) were boxed and labeled accordingly. Bar graphs of iTreg cells (D) and effector Treg cells (E) were derived accordingly. *n* = 6 biological replicates; *, *p* < 0.05, **, *p* < 0.01 (Student’s *t* test).

We then analyzed the population of CD4^+^CD25^+^Foxp3^+^ Treg cells in the scaffold-based 3D coculture model, where naïve CD4^+^ T cells were cocultured with primary IECs under inflammation and subsequently treated with EcN-gIFNL1. IL-2 is required for the proliferation and survival of CD4^+^CD25^+^Foxp3^+^ Treg cells, and low-dose exposure of IL-2 selectively promotes the expansion of CD4^+^CD25^+^Foxp3^+^ Treg cells in several different clinical studies.^40−42^ Therefore, we first measured and compared IL-2 concentrations in the supernatant under inflammation and under various treatments. Figure 3B shows that IL-2 concentrations from EcN-gIFNL1 or EcN-gYebF treatments were 40% lower than no treatment. A lower IL-2 concentration in the supernatant from EcN treatments suggests higher iTreg cell and effector Treg cell populations. Our flow cytometry results (Figure 3C–E) show a significant increase in the population of both iTreg cells (CD4^+^CD25^+^Foxp3^+^) and effector Treg cells (CD4^+^CD25^hi^Foxp3^hi^) from EcN-gIFNL1 treatment compared to no treatment but with inflammation. The reduction in IL-2 concentration correlates with the increase in both iTreg and effector Treg cell populations in the inflamed cocultures with EcN-gIFNL1 treatment, where low dose IL-2 was observed to enhance and maintain Treg cell populations.^43,44^ Depending on environmental cues, effector Treg cells are further differentiated from iTreg cells and they produce a high amount of immunosuppressive molecules such as IL-10.^45^ Furthermore, these observations also correlate with previous reports that Foxp3 negatively regulates the expression of IL-2.^46^

### EcN-gIFNL1 Suppresses the Expression of Pro-inflammatory Cytokines

Following the confirmation of the upregulation of CD4^+^CD25^+^Foxp3^+^ Treg cells (Figure 3) and the reduction of pro-inflammatory cytokines (Figure S4), we sought to confirm the concentrations of the various cytokines in the scaffold-based 3D coculture model (Table S2). EcN-gIFNL1 treatment significantly reduced the concentration of Th2 pro-inflammatory cytokines (IL-4, -5, -13, and -33), among which IL-33 was reduced to 33% compared to the control without EcN-gIFNL1 treatment. Recently, IL-33 was discovered to be a pro-inflammatory cytokine highly produced by epithelial cells, and it has been implicated in the development and pathology of UC via the IL-4 pathway *in vivo*.^47^ Furthermore, IL-33 was found to drive the differentiation of naïve helper T cells to Th2 cells as well as type II innate lymphoid cells (ILCs).^48,49^ IL-33 was upregulated by 3-fold in the inflamed coculture model without treatment. Upon EcN-gIFNL1 treatment, it was significantly reduced to a level similar to the uninflamed control. We did not observe a significant reduction of IL-33 upon EcN-gYebF treatment (Table S2).

**Figure 4:**
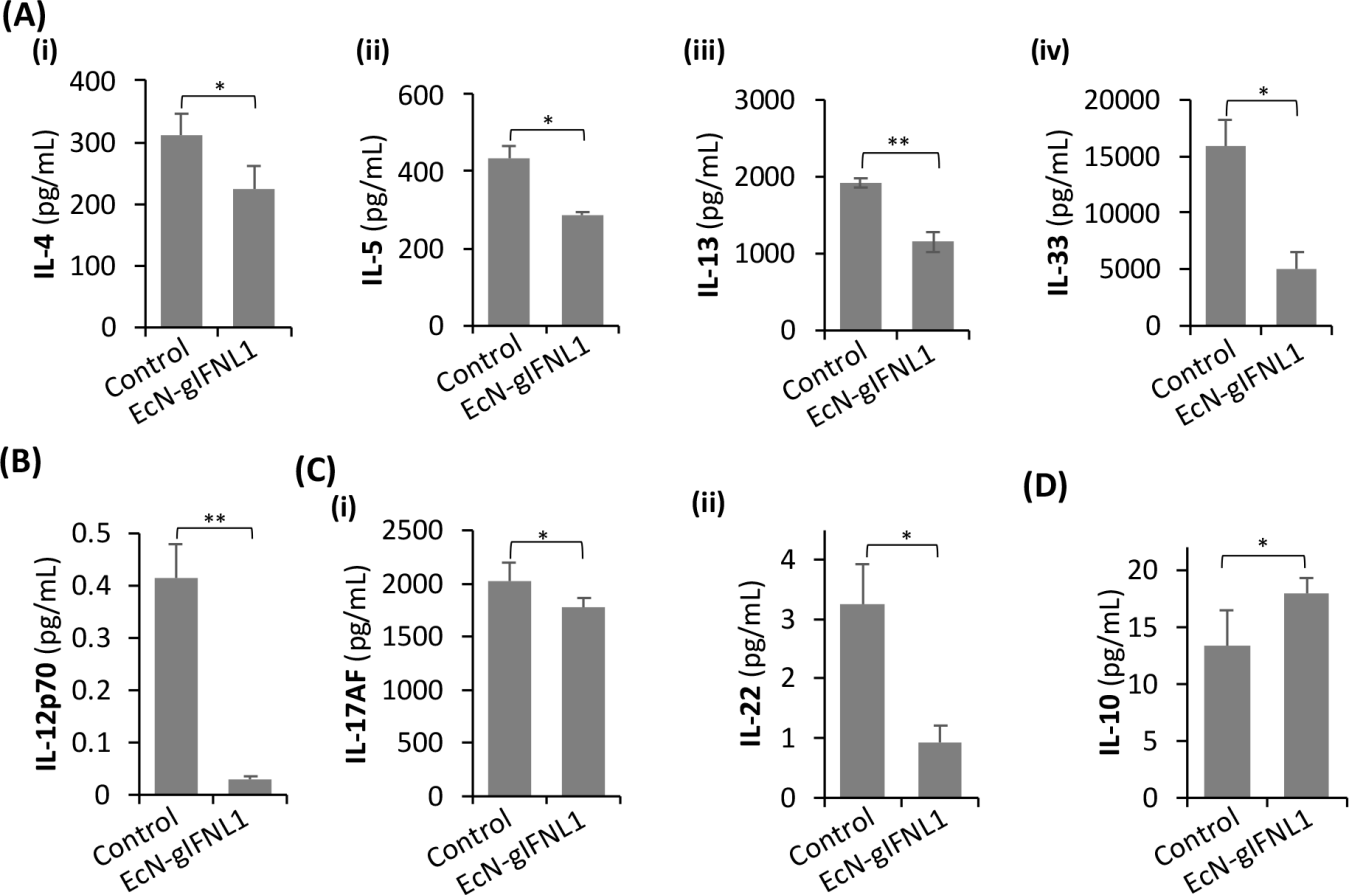
Regulation of Th1-, Th2-, and Th17-related pro-inflammatory cytokines by EcN-gIFNL1. Th2-related (A,i–iv), Th1-related (B), and Th17-related (C,i,ii) cytokines expressions in primary CD4^+^ T cells were significantly downregulated upon treatment with EcN-gIFNL1. (D) IL-10 expression was upregulated. *n* = 6 biological replicates; *, *p* < 0.05, **, *p* < 0.01 (Student’s *t* test).

Although IFNL1 was previously found to modulate Th1/Th2 cytokine expression by promoting Th1 cytokine production and suppressing Th2 cytokine expression,^50,51^ we observed a reduction in Th1 cytokine IL-12p70 upon EcN-gIFNL1 treatment (Figure 4B). The upregulation of IL-12p70 in the inflamed and untreated model is likely due to the presence of IFNγ while inducing inflammation. Although IL-12 is often implicated in the pathology of CD, it was also found to be upregulated in the serum and intestinal samples of UC patients.^52^ In addition to the downregulation of Th1 and Th2 pro-inflammatory cytokines, we also observed the significant downregulation of Th17-related cytokines (IL-17AF and -22) upon EcN-gIFNL1 treatment (Figure 4C). Treg and Th17 cells develop from naïve CD4^+^ T cells and can be induced by the same cytokine, TGFβ, although the environment determines into which subtypes naïve CD4^+^ T cells develop.^53^ Th17 cells are heavily implicated in various autoimmune diseases, and Treg cells are often dysregulated in autoimmune diseases.^54^ IL-17AF and IL-22 are the two most common IL-17 cytokines, which are pro-inflammatory and highly produced by Th17 cells. While IL-17AF levels did not increase by inflammation, EcN-gIFNL1 treatment caused a slight but significant decrease of IL-17AF. IL-22 is a pleiotropic cytokine that can be either pro- or anti-inflammatory, depending on the environment.^55,56^ We observed a significant increase in IL-22 levels in the inflamed coculture model. When the inflamed model was treated with EcN-gIFNL1, IL-22 levels were significantly reduced and comparable to the uninflamed control cells. This result suggests EcN-gIFNL1 ameliorates inflammation partially by reducing IL-22 levels, which has been observed to be higher in both murine colitis models and clinical IBD samples compared to healthy controls.^57^

Furthermore, we observed a slight increase in the IL-10 levels of inflamed models treated with EcN-gIFNL1 (Figure 4D), despite significant increases of iTreg and effector Treg cell populations (Figure 3). The slight increase of IL-10 is likely attributed to the possible reduction of IL-10-producing T-helper cells^58^ or IL-10 production in Treg cells in the presence of IFNL1. Nevertheless, our results indicate that EcN-gIFNL1 reduced pro-inflammatory cytokine production and suppressed the expression of Th2, Th1, and Th17 pro-inflammatory cytokines. Collectively, these results support the activity of the EcN-gIFNL1 toward ameliorating the inflammation associated with IBD development.

### Protective Effect of EcN-gIFNL1 on the Colorectal Epithelial Layer

**Figure 5:**
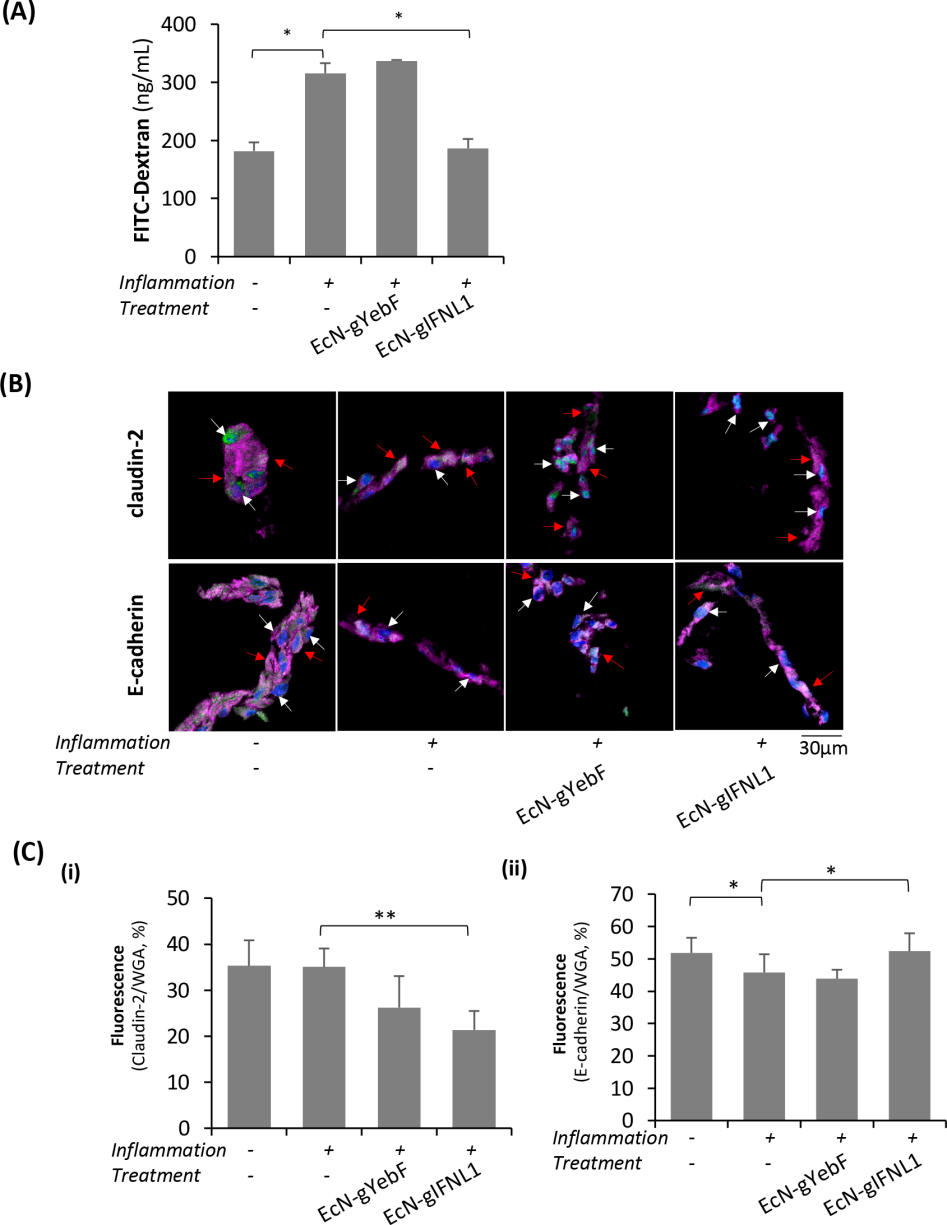
Protective effects of EcN-gIFNL1 on an inflamed epithelial tight junction. (A) Permeability of the intestinal epithelial cell (IEC) layer determined by the 10 kDa FITC-dextran assay. (B) Claudin-2 and E-cadherin were stained in green, and their localization was determined through the transverse sectioning of the IEC layer by confocal microscopy. White arrows indicate nuclei positions, while red arrows indicate the cell membrane (WGA, magenta). The blue color represents nuclei, and magenta represents stained cell membranes and cytosol. (C) The fluorescence intensity of claudin-2 (i) and E-cadherin (ii) in IECs was quantified from at least eight different fields from each sample.

Given the anti-inflammatory effects of EcN-gIFNL1, we hypothesized that EcN-gIFNL1 treatment might promote the integrity of the inflamed epithelial cell layer. We carried out permeability assays to confirm the effects of EcN-IFNL1 on inflamed epithelial cells. Figure 5A shows that EcN-gIFNL1 treatment cultures had 41% less FD10 in the basal compartment than the inflamed control without EcN treatment or with EcN-gYebF treatment, suggesting lower permeability of the inflamed epithelial cell layer and improved tight junctions with EcN-gIFNL1 treatment. We noted that the FITC-dextran concentration with inflammation and EcN-gIFNL1 treatment was comparable to the uninflamed control. EcN-gYebF treatment did not improve epithelial layer integrity, similar to the EcN-WT treatment (Figure 1E), confirming that EcN-gIFNL1, and not EcN-WT, confers a protective effect on the inflamed epithelial cell tight junctions.

To further confirm the protective effects of EcN-gIFNL1 on the inflamed epithelial tight junctions, we examined the localization and expression of two representative tight junction proteins, namely, claudin-2 and E-cadherin in IECs in transverse sections by confocal microscopy. Figure 5B shows that more claudin-2 (green) was localized to the cellular membrane (red arrows) in the inflamed IECs compared to the uninflamed control without EcN treatment. We also observed that more claudin-2 was localized to the nucleus (white arrows) in the inflamed IECs with EcN-gIFNL1 treatment compared to the controls with or without EcN-gYebF treatment. Figure 5B also shows that more E-cadherin (green) was localized to the cellular membrane in the inflamed cells with EcN-gIFNL1 treatment than the controls with or without EcN-gYebF treatment (Figure S7A). The fluorescence intensity of claudin-2 with EcN-gIFNL1 treatment was 40% lower than the inflamed control, suggesting the downregulation of claudin-2 production after EcN-gIFNL1 treatment (Figure 5C-i). The fluorescence intensity of E-cadherin with EcN-gIFNL1 treatment was slightly higher than the inflamed control, suggesting that EcN-gIFNL1 treatment rescued E-cadherin expression (Figure 5C-ii). Figure S7B shows the top-down view of the differential translocation of the proteins. The upregulation of claudin-2 expression may lead to increased paracellular transport of water and cations.^59^ Furthermore, claudin-2 expression is dependent on the presence of other pro-inflammatory cytokines such as IL-13, -17, -22, and TNF-α. Thus, claudin-2 upregulation is heavily implicated in various diseases of the small and large intestines.^60−62^ In contrast, the downregulation or complete loss of E-cadherin is a requirement of metagenesis, which can destabilize epithelial monolayers^63^ or cause cell deaths.^64,65^ In inflamed IECs with EcN-gIFNL1 treatment, reduced claudin-2 expression (Figure 5C-i) and increased E-cadherin expression (Figure 5C-ii) may contribute to maintaining the proper localization of other tight junction proteins, including E-cadherin and consequently resulting in a lower permeability (Figure 5A). These results are consistent with the upregulation of tight junction proteins (E-cadherin, occludin, and tricellulin) in inflamed Caco-2 cells with EcN-IFNL1 treatment (Figures 1F and S4), which contributed to protecting epithelial barrier integrity. Overall, our results support our hypothesis that EcN expressing IFNL1 protects epithelial tight junctions by modulating the localization and expression of tight junction proteins.

## Conclusions

**Figure 6:**
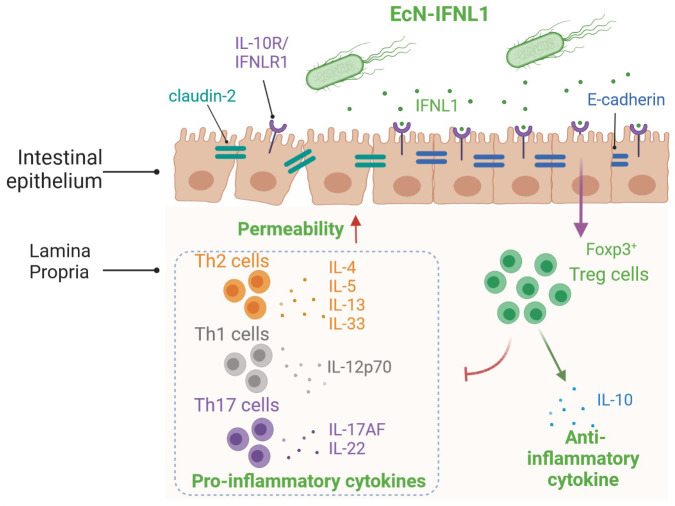
Proposed anti-inflammatory and immunomodulatory mechanisms of EcN-gIFNL1. In an inflamed environment, pro-inflammatory cytokines are secreted and epithelial permeability is increased. EcN-gIFNL1 secretes the cytokine IFNL1 in such inflamed environments. Upon binding with its receptor on epithelial cells, a cascade of downstream signaling is activated. One such event includes the induction of transcription factor Foxp3 in naïve CD4^+^ T cells in the lamina propria, promoting the T cells’ differentiation into iTreg cells. The iTreg cells in turn suppress different T helper (Th) cells, which were drawn to the epithelial barrier upon inflammation. Our data has shown the significant suppression of pro-inflammatory cytokines expression by these Th cells. Besides immunomodulation, EcN-gIFNL1 can reduce the expression of the channel protein claudin-2 and restore the expression of the tight junction protein E-cadherin.

In this study, we engineered EcN strains that expressed and secreted about 6 ng/mL IFNL1 upon NO induction. Though primarily known as an antiviral cytokine, IFNL1 (at 300 ng/mL) can activate the JAK/STAT and MAPK signaling pathways that typically control the expression of pro-inflammatory cytokines.^66^ Our study, however, shows that, in an active inflammatory environment, treatment with the engineered strain EcN-gIFNL1 reduced levels of pro-inflammatory cytokines (related to Th1, Th2, and Th17 cells) and increased levels of the anti-inflammatory cytokine IL-10 as well as iTreg and effector Treg cell populations. It also improved the expression of tight junction proteins (e.g., E-cadherin, occludin, tricellulin), reduced claudin-2 expression, maintained tight junction protein localization, and thereby ameliorated intestinal permeability dysfunction in the inflamed epithelial layer in an *in vitro* cell line and in 3D scaffold coculture models. Accordingly, we proposed the mode of action of EcN-gIFNL1 in ameliorating inflammation in our IBD models (Figure 6). To our knowledge, our work represents the first study that engineered a probiotic bacterium to express and secrete IFNL1 in a controllable manner, demonstrating its anti-inflammatory effects in *in vitro* IBD models. Our study describes a potentially new route for delivering IFNL1 as a therapeutic agent in a probiotic chassis, providing an alternative to *IFNL1* gene transfer via recombinant adenovirus.^11,67^ As a generally regarded as safe (GRAS) substance,^68^ the development of EcN as a chassis for delivering IFNL1 to the inflamed colorectal epithelium is more applicable than delivery by other means, including through adenovirus, which is unstable and restricted to gene transfer. Future studies can focus on validating the efficacy of EcN-gIFNL1 in murine IBD models as well as clinical IBD samples.

## Supporting Information Available

Quantification of Western blot bands in Figure 1F; concentration of cytokines in Figure 4; schematics of scaffold-based 3D coculture model and transverse sectioning of cells grown on a transwell membrane; effect of sodium nitroprusside on cell growth and nitrite generation; expression of *iNOS* gene, pro-inflammatory cytokines, and tight junction proteins in inflamed Caco-2/Jurkat T cells coculture; flow cytometric analysis of CD4^+^ T cells cultured with only EcN-gIFNL1 in an inflammatory environment; immunofluorescent images of top-down view of the epithelial cells in the 3D coculture model (PDF)

## Abbreviations

CD: Crohn’s disease
EcN: *E. coli* Nissle 1917
FC: fold change
10 kDa FITC-dextran: FD10
GFP: green fluorescence protein
*gIFNL1*: genomically integrated *IFNL1*
*gYebF*: genomically integrated *YebF*
IBD: inflammatory bowel disease
IEC: intestinal epithelial cell
IFNL: interferon lambda
IFNLR1: IFNL1 receptor 1
IL: interleukin
NO: nitric oxide
iNOS: inducible nitric oxide synthase
rhIFNL1: recombinant human IFNL1
SNP: sodium nitroprusside
Th cells: T helper cells
Treg cell: regulatory T cells
UC: ulcerative colitis
WT: wild-type
