# Supplementary material for: An Engineered Probiotic Produces a Type III Interferon IFNL1 and Reduces Inflammations in in vitro Inflammatory Bowel Disease Models
Source: ACS Biomater Sci Eng. 2022 Nov 18;9(9):5123–35. doi: 10.1021/acsbiomaterials.2c00202 (PMC10498420; doi:10.1021/acsbiomaterials.2c00202)
Supplement: Supplementary file 1 — ab2c00202_si_001.pdf [file ab2c00202_si_001.pdf]

An Engineered Probiotic Produces a Type III Interferon IFNL1 and Reduces Inflammations in *in vitro* Inflammatory Bowel Disease Models

Koon Jiew Chua [1,2,3,4], Hua Ling [1,2,3,4*#], In Young Hwang [1,2,3,4§], Hui Ling Lee [1,2,3,4], John C. March[5], Yung Seng Lee[1,2,6], Matthew Wook Chang [1,2,3,4*]

[1] NUS Synthetic Biology for Clinical and Technological Innovation (SynCTI), National University of Singapore, 117456, Singapore
[2] Synthetic Biology Translational Research Programme, Yong Loo Lin School of Medicine, National University of Singapore, 117456, Singapore
[3] Department of Biochemistry, Yong Loo Lin School of Medicine, National University of Singapore, 117596, Singapore
[4] Wilmar-NUS Corporate Laboratory, National University of Singapore, 117599, Singapore
[5] Department of Biological and Environmental Engineering, Cornell University, Ithaca, New York 14853, USA
[6] Department of Paediatrics, Yong Loo Lin School of Medicine, National University of Singapore, Singapore 119228, Singapore

Present addresses
[#] HL: Wilmar Innovation Centre, Wilmar International Limited, 28 Biopolis Road, Singapore 138568
[§] IYH: Singapore Institute of Technology, 10 Dover Dr, Singapore 138683

Supplementary Information

Supplementary Table

Table S1. Quantification of relative band intensity of proteins under inflammation as analyzed through a Western blot

Table S2. Concentration of cytokines from scaffold-based 3D co-culture model

Supplementary Figures

Figure S1. Schematics of the scaffold-based 3D co-culture model and transverse sectioning of cells grown on a transwell membrane

Figure S2. Effect of sodium nitroprusside to cell growth and nitrite generation

Figure S3. Expression level of *iNOS* gene under inflammation in inflamed Caco-2 cells co-cultured with Jurkat T cells

Figure S4. Multiplex assay of pro-inflammatory cytokines in Caco-2 cells co-cultured with Jurkat cells and EcN-IFNL1

Figure S5. Expression level of tight junction proteins in Caco-2 cells co-cultured with EcN-IFNL1

Figure S6. Flow cytometric analysis of the effect of EcN-gIFNL1 on enhancing the population of CD4+CD25+Foxp3+ induced Treg (iTreg) cells.

Figure S7. EcN-gIFNL1 influences tight junction protein localization

Supplementary Tables

Table S1. Quantification of relative band intensity of proteins under inflammation as analyzed through a Western blot*

	Treatment			
**Protein**	**Control**	**rhIFNL1**	**WT EcN**	**EcN-IFNL1**
E-cadherin	0.768816	0.822359	1.032248	1.356463
occludin	0.683316	1.062393	1.310284	1.71344
tricellulin	0.683041	1.682621	2.41368	2.765025
claudin-2	1.249857	0.768601	0.551975	0.499847
β-actin	1.003481	1.025454	1.04466	1.254325

* Protein intensity uninflamed was set to 1. Control, without IFNL1 nor EcN treatments.

Table S2. Concentration of cytokines (pg/mL) from scaffold-based 3D co-culture model

**Cytokines**	**Uninflamed**	**Inflamed**		
		**Untreated**	**EcN-gYebF**	**EcN-gIFNL1**
IL-4	281.86 ± 35.49	310.46 ± 35.95	203.07 ± 14.45	224.85 ± 27.02
IL-5	353.42 ± 13.45	434.49 ± 30.11	387.84 ± 74.01	286.115 ± 6.78
IL-10	7.26 ± 0.38	13.26 ± 3.11	26.907 ± 1.96	17.985 ± 1.35
IL-12p70	0.11 ± 0.01	0.415 ± 0.06	0.145 ± 0.05	0.03 ± 0.01
IL-13	1580.115 ± 93.5	1924.1 ± 60.8	1484.31 ± 174.63	1151.175 ± 135.12
IL-17AF	1977.015 ± 39.72	2028.9725 ± 174.1	2271.235 ± 231.33	1778.1925 ± 92.68
IL-22	1.025 ± 0.46	3.25 ± 0.67	1.875 ± 0.32	0.9275 ± 0.29
IL-33	6892 ± 767.79	15961.53 ± 2293.93	12895.035 ± 2042.75	4966.03 ± 1503.01

Supplementary Figures

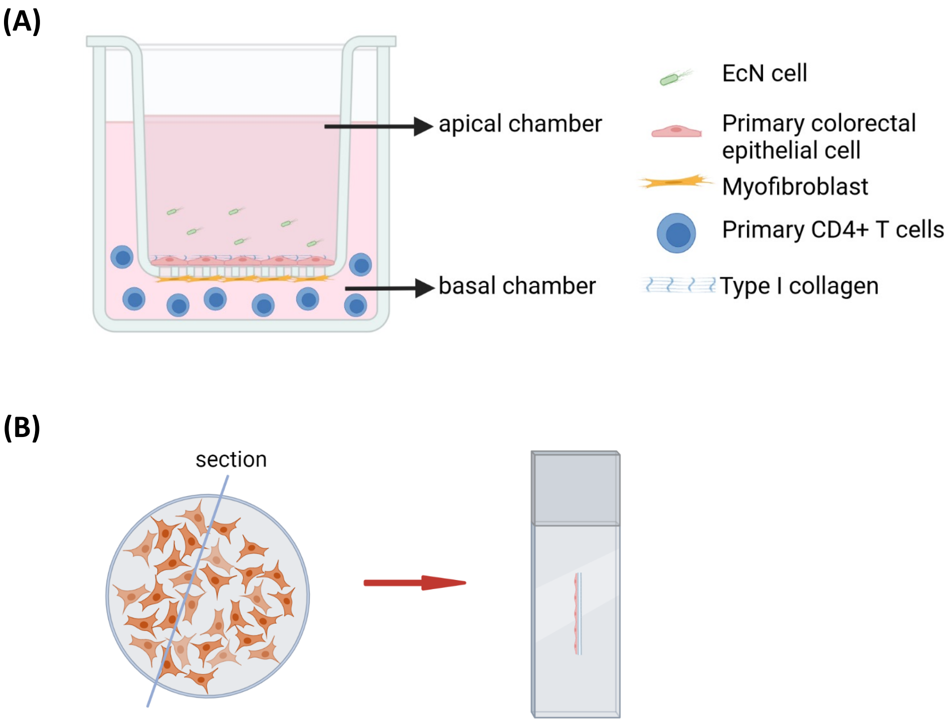

Figure S1. Schematics of the scaffold-based 3D co-culture model and transverse sectioning of cells grown on a transwell membrane. (A) Schematic of the scaffold-based 3D co-culture model. The apical side of the transwell membrane was first coated with type I collagen to form a matrix, followed by the seeding of primary epithelial cells. Myofibroblasts were then seeded on the basal side of the membrane. Naïve CD4+ T cells were cultured in the basal compartment 5 d after the seeding of epithelial cells and myofibroblasts. Inflammation agents were then added to the apical compartment. After 36 h inflammation, EcN cells were added to the apical compartment for 10 h. (B) Schematic of transverse sectioning of cells grown on a transwell membrane. The membrane was excised carefully from the plastic holder and cryofixed before sectioning. Each section was then fixed on a glass slide, after which it was visualized under confocal microscope.

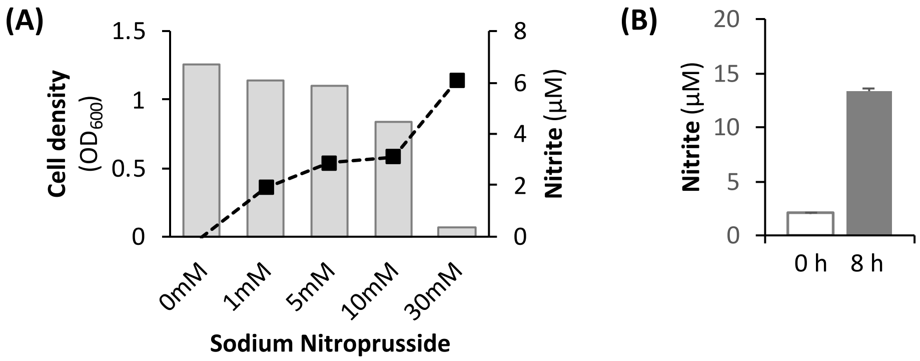

Figure S2. Effect of sodium nitroprusside to cell growth and nitrite generation. (A) Sodium nitroprusside (SNP) at various concentrations was supplemented to EcN cells for 24 h. Cell density was determined and nitrite concentration in the supernatants were obtained using the Griess method. (B) Amount of nitrite was determined after 8 h of SNP supplementation to culture media. n = 5.

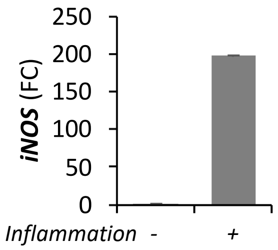

Figure S3. Expression level of *iNOS* gene under inflammation in inflamed Caco-2 cells co-cultured with Jurkat T cells. FC, fold change.

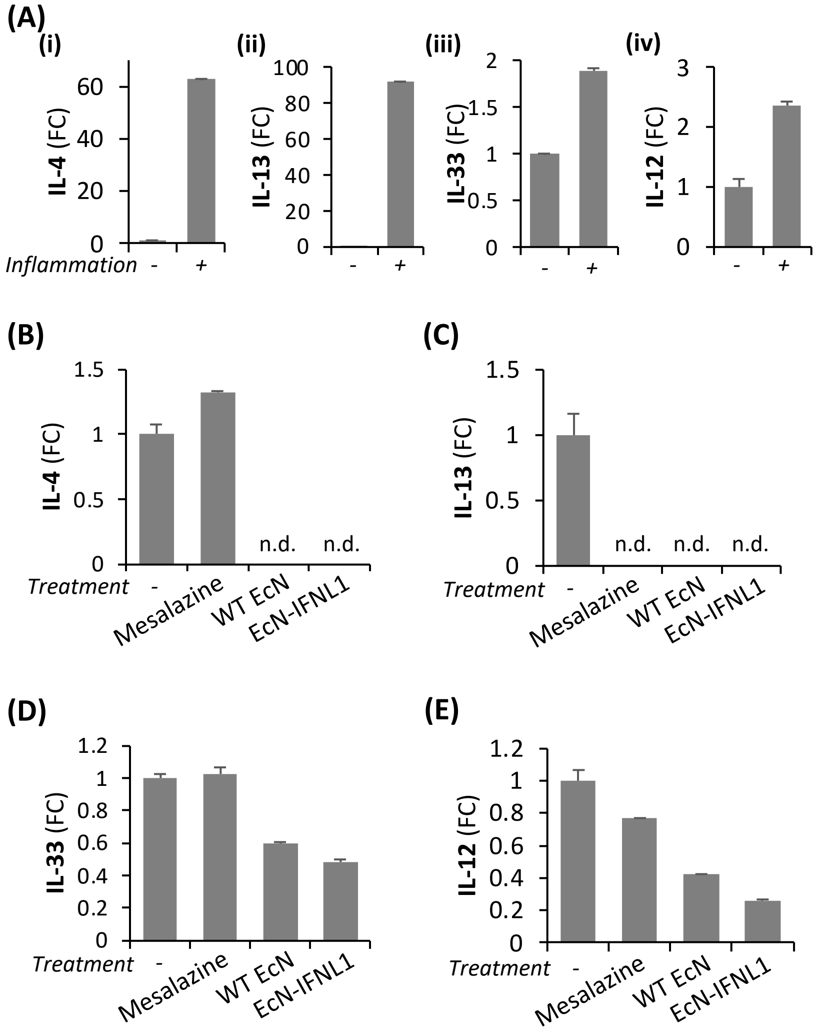

Figure S4. Multiplex assay of pro-inflammatory cytokines in Caco-2 cells co-cultured with Jurkat cells and EcN-IFNL1. (A) Concentrations of pro-inflammatory cytokines was confirmed in inflamed co-culture of Caco-2/Jurkat T cells. IL-4 (B), IL-13 (C), IL-33 (D) and IL-12 (E) were significantly downregulated by EcN-IFNL1. FC, fold change.

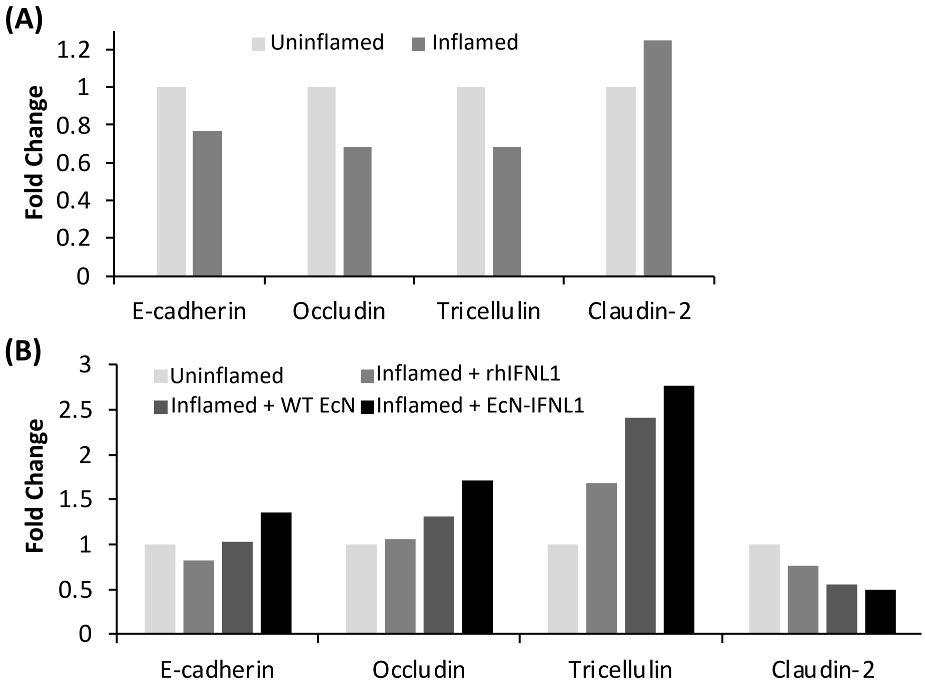

Figure S5. Expression level of tight junction proteins in Caco-2 cells co-cultured with EcN-IFNL1. Fold changes of various tight junction proteins without treatment (A) and with treatments, including rHINFL1, EcN, and EcN-INFL1 (B) by quantifying the western blot results (Fig. 1D) are shown.

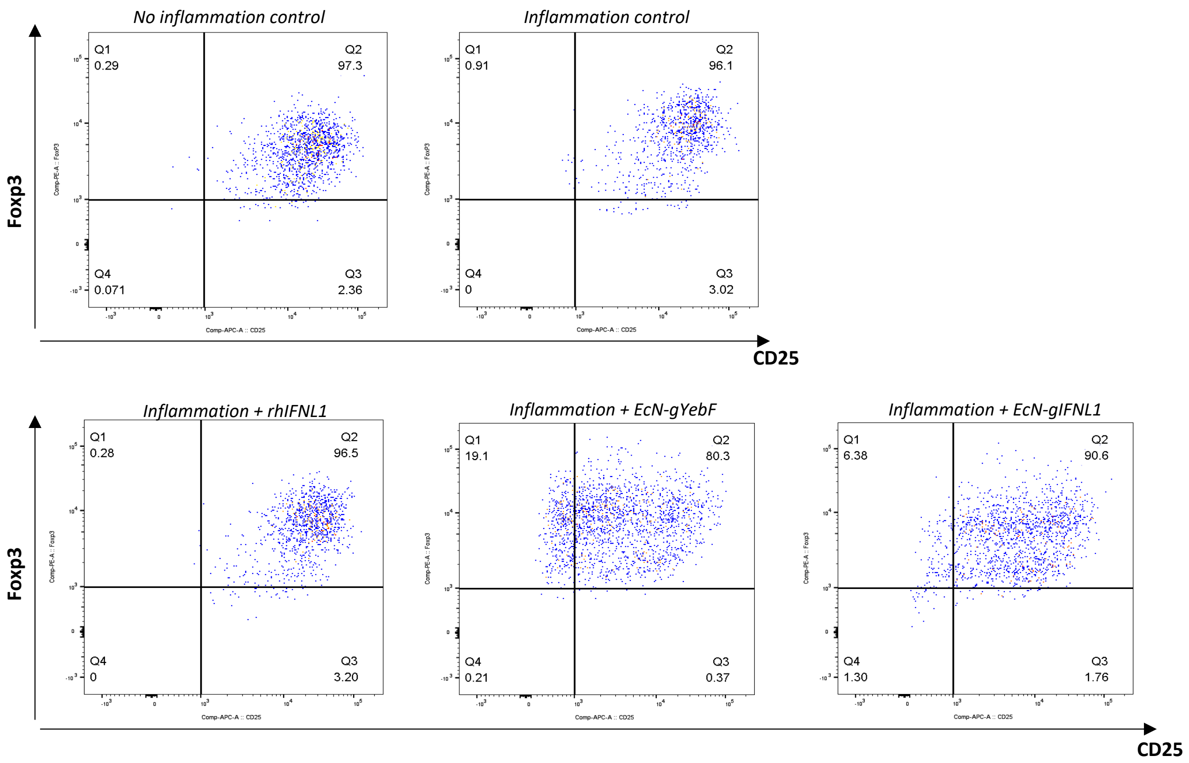

Figure S6. Flow cytometric analysis of the effect of EcN-gIFNL1 on enhancing the population of CD4$^+$CD25$^+$Foxp3$^+$ induced Treg (iTreg) cells. The dot plots of flow cytometry are shown.

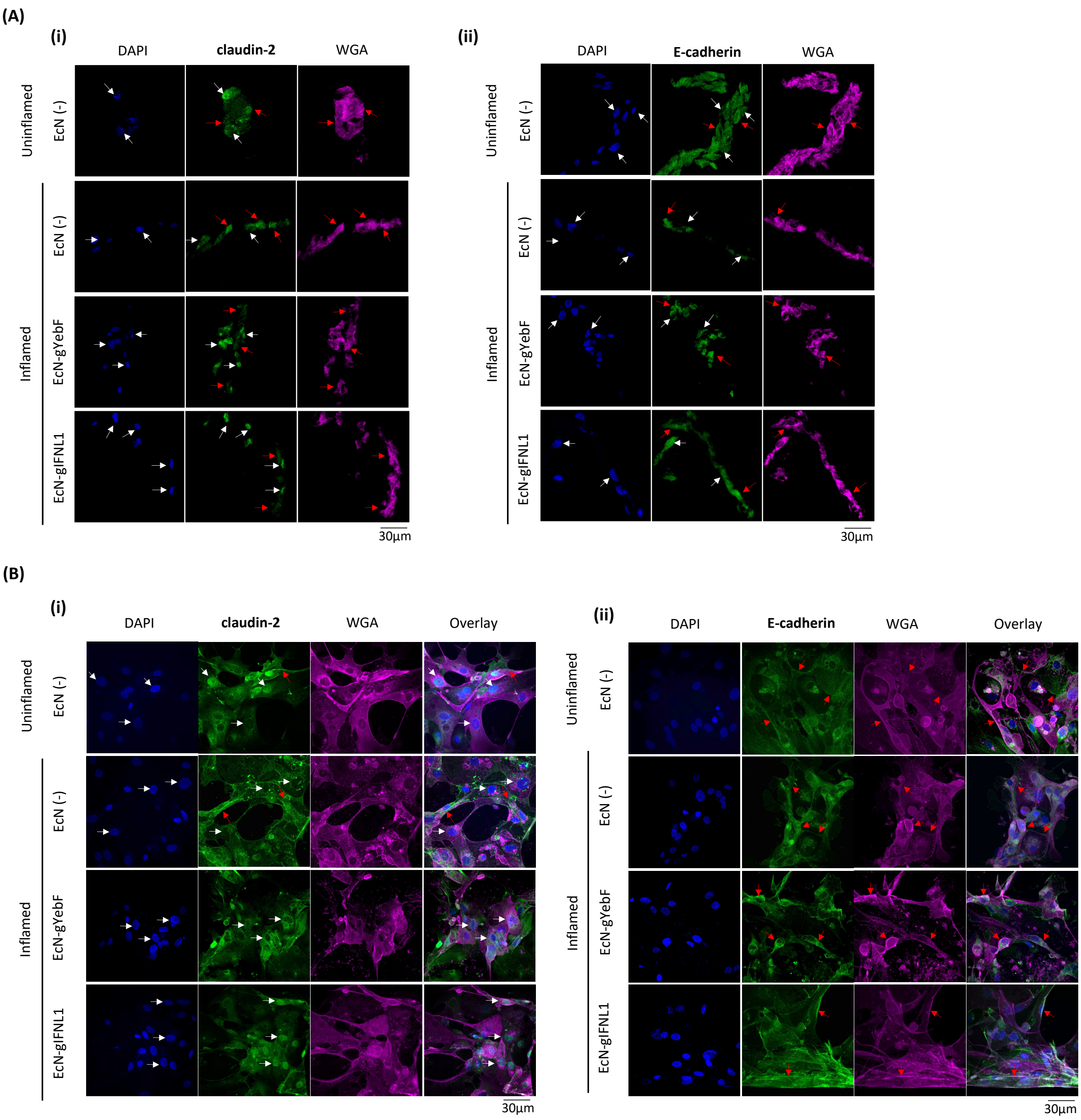

Figure S7. EcN-gIFNL1 influences the localization of proteins at tight junctions. (i) claudin-2 and (ii) E-cadherin were stained in green and their localization was determined through (A) transverse views before overlay as shown in Fig. 5, and top-down views (B) of the IECs layer. White arrows indicate nuclei positions (DAPI, blue), while red arrows indicate the cell membrane (WGA, magenta).
